# Cu_2+1_O coated polycrystalline Si nanoparticles as anode for lithium-ion battery

**DOI:** 10.1186/s11671-016-1426-5

**Published:** 2016-04-22

**Authors:** Junying Zhang, Chunqian Zhang, Shouming Wu, Zhi Liu, Jun Zheng, Yuhua Zuo, Chunlai Xue, Chuanbo Li, Buwen Cheng

**Affiliations:** State Key Laboratory on Integrated Optoelectronics, Institute of Semiconductors, Chinese Academy of Sciences, Beijing, 100083 China; Zhejiang Fluoride and Silicon Research Institute, Quzhou, Zhejiang 324100 China

**Keywords:** Polycrystalline Si, Cu_2+1_O, Composite anode, Lithium-ion battery

## Abstract

Cu_2+1_O coated Si nanoparticles were prepared by simple hydrolysis and were investigated as an anode material for lithium-ion battery. The coating of Cu_2+1_O on the surface of Si particles remarkably improves the cycle performance of the battery than that made by the pristine Si. The battery exhibits an initial reversible capacity of 3063 mAh/g and an initial coulombic efficiency (CE) of 82.9 %. With a current density of 300 mA/g, its reversible capacity can remains 1060 mAh/g after 350 cycles, corresponding to a CE ≥ 99.8 %. It is believed that the Cu_2+1_O coating enhances the electrical conductivity, and the elasticity of Cu_2+1_O further helps buffer the volume changes during lithiation/delithiation processes. Experiment results indicate that the electrode maintained a highly integrated structure after 100 cycles and it is in favour of the formation of stable solid electrolyte interface (SEI) on the Si surface to keep the extremely high CE during long charge and discharge cycles.

## Background

The application of lithium-ion battery is playing an important role in the development of portable electric and electro vehicles. Graphite has dominated as the anode material of lithium-ion batteries and has been commercialized for many years due to its excellent behaviour during prolonged charge/discharge cycles. However, the theoretical capacity of graphite is limited to 372 mAh/g, which is low relative to the requirement of high energy density application fields [1]. To develop a low-cost electrode material with a high energy capacity is of great significance to improve the performance of products that use rechargeable batteries. Crystal Si has attracted much attention as a possible anode candidate due to the much higher lithium storage capacity (4200 mAh/g, about ten times higher than graphite), low lithium alloying/dealloying potential, long discharge plateau and natural abundance [2]. However, Si-based anodes also face grand challenges due to the large volume expansion (about 400 %) of the Si particles during lithiation/delithiation processes. It results in pulverization, breaks the electrical contact of the electrode structure and brings in great capacity decay [3]. The lack of electrical contacts between Si particles or between Si and current collector even makes capacity fading worse. Many investigations have been done to accommodate this severe volume expansion, mainly including novel nanostructured Si such as Si wires [4], Si tubes [5], porous Si thin films [6] and nest-like Si nanospheres [7] or multiphase composites consisting of active Si and other active/inactive phases [8, 9]. Among these materials, Si-based composites containing Si and coating other ductile materials as buffer were conducted to reduce volume expansion. Carbon, metal, metal oxide and conducting polymers are used as the shell materials, which can act as both conducting and mechanical supporting material [10–14]. Among them, the coating of metal and metal oxide on Si electrodes will be a good way to improve their electrochemical performances. Due to the introduction of the surplus metal Cu in the coating layer, it has been proved to greatly increase the electrical conductivity in a Si-based anode system and help buffer the volume changes during insertion/extraction processes of lithium ions [15–17].

In this study, a scalable, chemical approach for synthesizing Cu_2+1_O-coated polycrystalline Si particles through a hydrolysis method is reported. The Cu_2+1_O-coated particles were utilized as anode material for lithium-ion battery. The coating of Cu_2+1_O on Si particles reduced charge transfer resistance, increased the reversible capacity and improved the tolerance for volume changes during lithiation/delithiation processes.

Our investigation revealed that Cu_2+1_O-coated Si electrode showed significantly improved cycle performance even after 350 cycles. It maintained an extremely integrated structure after 100 cycles. It is confirmed that the Cu_2+1_O coating on Si enhanced the conductivity and buffered the volume changes during insertion/extraction processes of lithium ions, leading to a highly stable cycle performance.

## Methods

### Experimental procedures

Si particles of 80 nm were treated by hydrofluoric acid (HF, 10 %) for 10 min and then centrifuged and washed by deionized water three times. Ammonium formate (6.3 g) was dissolved in deionized water (500 mL), and formic acid (0.5 mL) was added. After copper sulphate (0.007 mol/L) was added and dissolved, the Si particles treated by HF were added and the reaction was maintained at a constant temperature of 70 °C for 2 h. After that, the material was centrifuged and washed by deionized water three times and heated in vacuum drying oven overnight. Then, the composite particles, super P, graphite, VGCF and polyacrylic acid were mixed for 6:1:1:1:1 and stirred for 6 h to be evenly mixed. The slurry was coated on a copper foil with a thickness of 100 μm, followed by heat treatment at 110 °C in a vacuum drying oven overnight, and then, the anode was ready for cell assembling.

### Characterization

The morphologies of the pristine Si particles and Cu_2+1_O-coated Si particles were investigated by transmission electron microscope (TEM), which was performed with a small amount of sample dispersed in ethanol and dropped into Au TEM grid for analysis. The structures were characterized by X-ray diffraction (XRD) to find the crystallinity and composition of the sample, and the radial data (2θ) were integrated over 10°–70°.

### Electrochemical measurements

Coin-type half cells (2025R type) with lithium foils as counter electrodes were assembled in a glove box (Mikrouna Super 1220/750) under an argon atmosphere. The electrolyte used was LiPF_6_ (1 M) in ethylene carbonate-methyl ethyl carbonate (30:70 vol%), with vinylene carbonate and fluoroethylene carbonate as additives. Glass fibre was employed to stabilize the coin system. The coin-type half cells were galvanostatic cycled (with a constant current of 300 mAh/g and cut-off voltages of 0.01 and 1.2 V at 25 °C) on land battery test system. The cyclic voltammetry (CV) curves (in the range of 10 mV to 2.6 V and at a rate of 0.2 mV/s) and electrochemical impedance spectroscopy (EIS, in the range of 100,000 to 0.01 Hz at a magnitude of 0.05 mV) were measured with an electrochemical workstation (PGSTAT302N, Autolab).

## Results and discussion

**Fig. 1:**
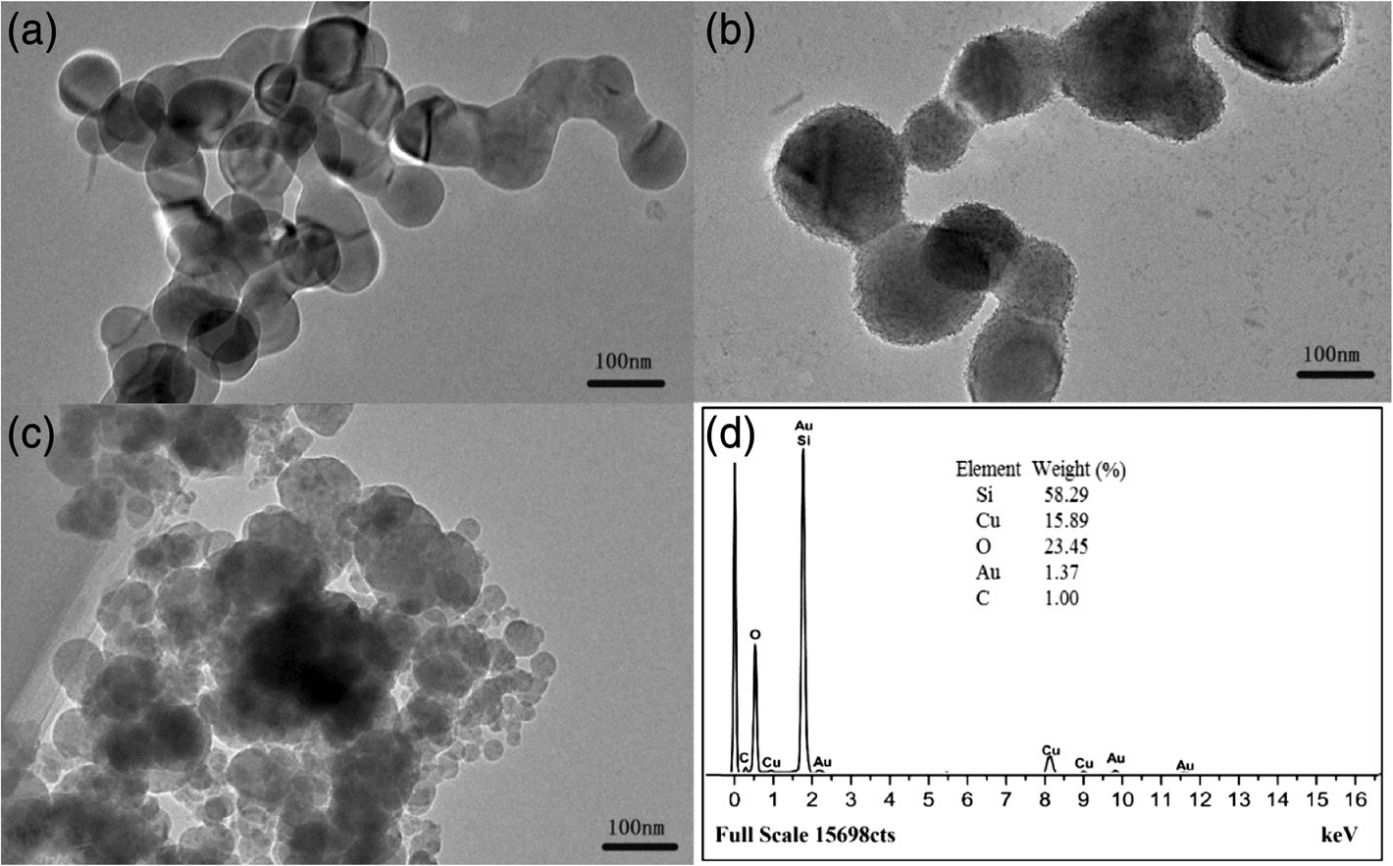
TEM images of **a** pristine Si particles, **b** HF treated Si particles and **c** Cu_2+1_O-coated Si particles. **d** EDS analysis of Cu_2+1_O-coated Si particles

Figure 1 shows the TEM images of the samples. Clearly, the pristine Si particles have a smooth surface with the size of about 80 nm as shown in Fig. 1a. Their surface becomes rough after HF treatment due to the reaction of HF with SiO_2_ on the surface of Fig. 1b. This surface roughness is proved to be beneficial for the adherence of Cu_2+1_O with Si during hydrolysis. It can be found that the nano-sized irregular-shaped Cu_2+1_O particles decorate on the Si sample surface after hydrolysis (Fig 1c). The energy dispersive X-Ray spectroscopy (EDS) examination confirms the existence of Si, Cu, O, Au and C in Cu_2+1_O-coated Si particles. Here, Au and C contents are from the TEM grid, Cu is from the deposition of Cu_2+1_O on the surface of HF treated Si particles and O is from both Cu_2+1_O and the remaining SiO_2_. The weight ratio of Cu_2+1_O and Si in the final composite is about 1:3.

**Fig. 2:**
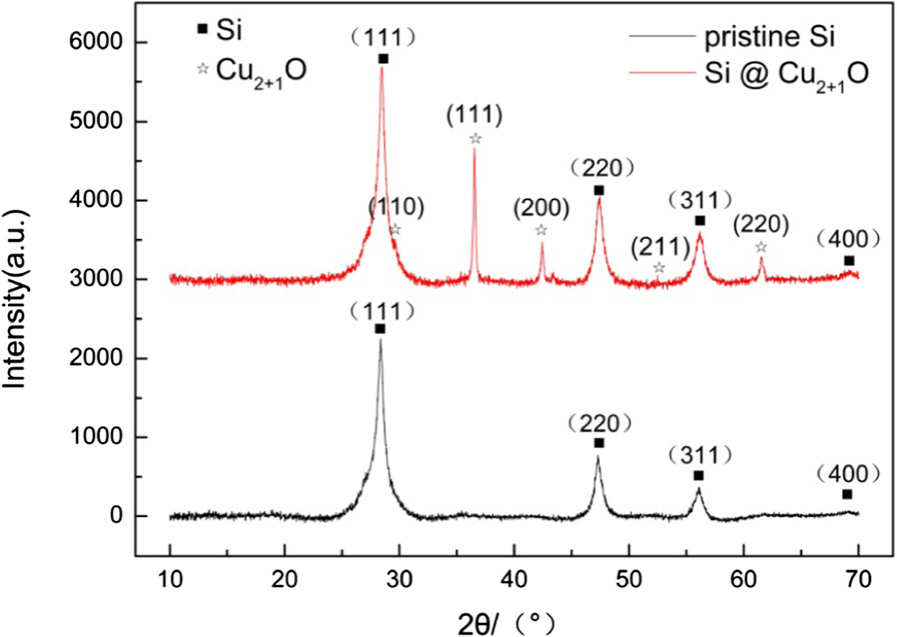
XRD of Cu_2+1_O-coated Si particles and pristine Si particles

XRD results confirm that the successful coating of Cu_2+1_O on the surface of Si particles by the chemical precipitation during the hydrolysis and drying process since all peaks marked in Fig. 2 match well with Bragg peaks of Si and Cu_2+1_O, and the peaks of Si are consistent with the peaks of pristine Si XRD results.

**Fig. 3:**
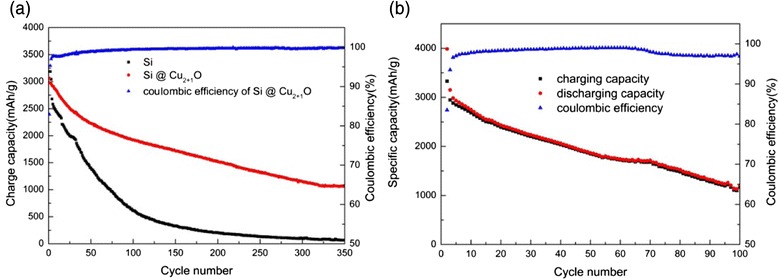
**a** Charge capacity of Cu_2+1_O-coated Si electrode and pristine Si electrode and CE of Cu_2+1_O-coated Si electrode. **b** Coulombic efficiency and specific capacity of Cu_2+1_O-coated Si electrode with current density of 800 mA/g

These materials were then used as the anode and assembled into a lithium-ion battery. Experimental results reveal that the coating of Cu_2+1_O on Si particles remarkably improved the cycle performance of the battery. It has an initial reversible capacity up to 3063 mAh/g and an initial coulombic efficiency (CE) of 82.9 % (Fig. 3a). With a current density of 300 mA/g, the reversible capacity of the composite electrode remains 62.8 % after 100 cycles, as 1923.5 mAh/g with a CE ≥99.7 %, and even after 350 cycles, the reversible capacity is more than 1060 mAh/g with a CE ≥99.8 %. As comparison, the reversible capacity of the composite electrode with 80-nm pristine Si which is purchased without any treatment decreases significantly, which remains only 19.2 % after 100 cycles, as 613.6 mAh/g with the same current density of 300 mA/g (Fig. 3a). What is more, the cycle performance of Cu_2+1_O-coated Si electrode with a higher current density of 800 mA/g is conducted. It has an initial reversible capacity up to 3329 mAh/g and an initial CE of 83.5 % (Fig. 3b). Even after 100 cycles, the reversible capacity was more than 1110 mAh/g with a CE ≥97 %. We have made the parallel tests for each cell, and the repeatability and consistency of the cell performance is good. These results reveal that the coating of Cu_2+1_O on Si as electrode has greatly improved cycle performance than pristine Si electrode. Compared to the pristine Si electrode, the high capacity retention and high CE of Cu_2+1_O coated Si electrode is because the coating of Cu_2+1_O enhances the conductivity and effectively suppresses the volume changes during insertion/extraction processes of lithium ions and helps the formation of stable solid electrolyte interface (SEI) [18, 19] .

**Fig. 4:**
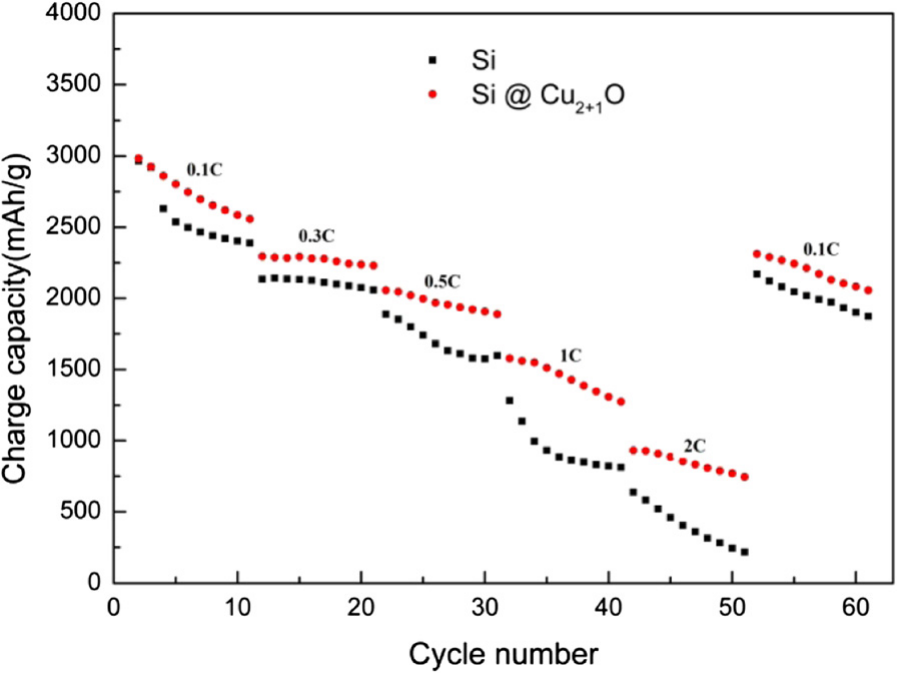
Charge capacity of Cu_2+1_O-coated Si electrode and pristine Si electrode at various current rates (1 C = 1000 mA/g)

In order to investigate the rate performance of Cu_2+1_O-coated Si electrode, galvanostatic measurements were conducted from 0.1 C (1 C = 1000 mA/g) to 2 C (Fig. 4) with current density of 0.1, 0.3, 0.5, 1, 2 and 0.1 C (each density of current for ten cycles). Obviously, the Cu_2+1_O-coated Si electrode has better rate performance with capacities of 2984.7, 2293.3, 2056.4, 1579.5 and 931.9 mAh/g at the first cycle of each current density. It rises again up to 2310.9 mAh/g at low current density (0.1 C). This may be related to the activation of the unreacted inner region of silicon, which is generally accepted for Si anodes operated under high rate conditions [20]. As comparison, pristine Si electrode has lower capacities of 2966.5, 2135.2, 1887.5, 1282.8 and 637.8 mAh/g, at the first cycle of each current density, and it rises back up to 2170.3 mAh/g at 0.1 C.

**Fig. 5:**
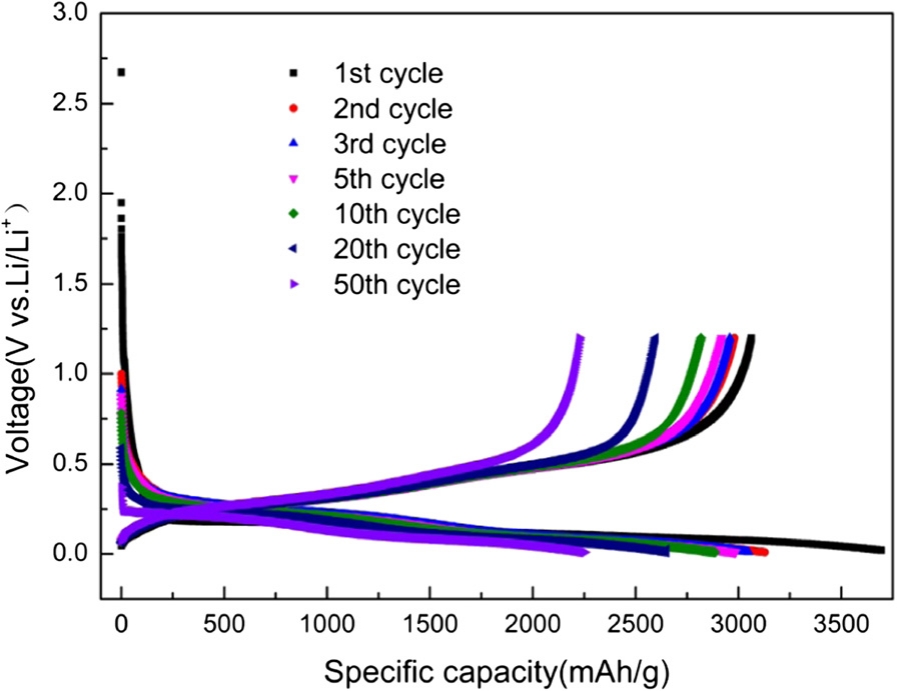
Voltage vs. capacity of battery with Cu_2+1_O-coated Si electrode

Figure 5 shows the capacity of Cu_2+1_O-coated Si electrode as the function of the voltage. The charge and discharge profiles of Cu_2+1_O-coated Si electrode for the first, second, third, fifth, tenth, 20th and 50th cycles are tested with a current density of 300 mA/g. The wide plateau at about 0.2 V in the discharge process is original from the formation of Li_x_Si alloy [21, 22]. The initial discharge and charge capacities are 3694.8 and 3063.1 mAh/g, respectively, with a CE of 82.9 %. During the second cycle, a large reversible capacity of 2979.9 mAh/g is obtained, with a CE of 95.3 %. Even during the 50th cycle, the reversible capacity still remains 2227.5 mAh/g, with a high CE of 99 %. The stable cycle performance is benefiting from the Cu_2+1_O coating layer, which contributes to the increased conductivity and suppression of volume changes of Si during insertion/extraction processes of lithium ions.

**Fig. 6:**
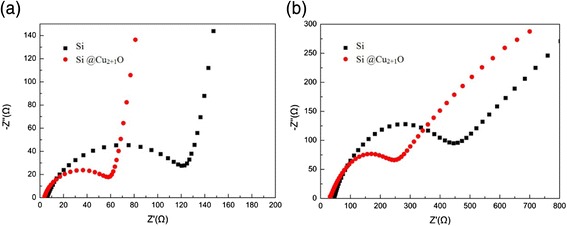
Impedance comparison of Cu_2+1_O-coated Si electrode and pristine Si electrode **a** uncycled and **b** after 100 charge and discharge cycles

In order to further elucidate the effect of Cu_2+1_O as conductive material, the impedance spectroscopies were investigated for the pristine Si and Cu_2+1_O-coated Si electrodes (as prepared and after 100 charge/discharge cycles), and the results are showed in Fig. 6. All of the electrodes used in the impedance test have the same thickness and area, which are 100 μm and π*(1.5/2)^2^ cm^2^, respectively, so only resistance is used for comparison. Apparently, the diameter of the semicircle for the Cu_2+1_O-coated Si electrode is smaller than that of the pristine Si before cycling, indicating the smaller surface resistance and charge transfer resistance as shown in Fig. 6a. Thereafter, the impedance test after 100 cycles indicates that pristine Si shows a larger charge transfer resistance as shown in Fig. 6b. The possible reason is that lithium ion conduction in the SEI layers and charge transfer at the electrode/electrolyte interface are hindered by the increased defects due to the volume changes and structure failure during continued cycling. In contrast, the semicircle, corresponding to the Cu_2+1_O-coated Si, shows increased but lower charge transfer resistance after 100 cycles as also shown in Fig. 6b. This indicates the Cu_2+1_O deposited on the Si surface is beneficial for the formation of low resistance SEI layer [23].

**Fig. 7:**
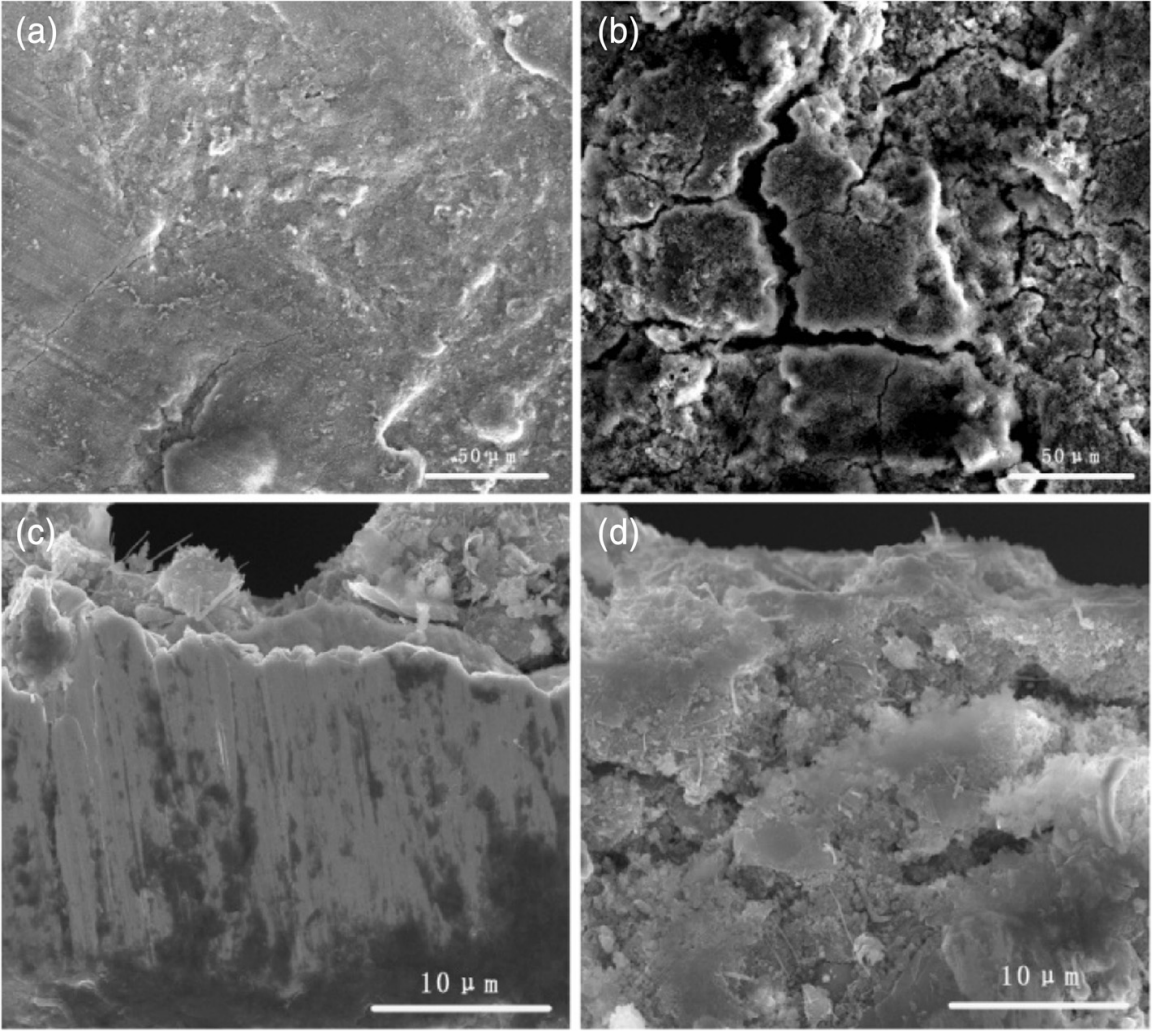
SEM images of the surface of **a** Cu_2+1_O-coated Si electrode, **b** pristine Si electrode, cross section of **c** Cu_2+1_O-coated Si electrode and **d** pristine Si electrode after 100 charge and discharge cycles

The surface and the cross section SEM images of Cu_2+1_O-coated Si electrode and pristine Si electrode after 100 charge and discharge cycles are showed in Fig. 7. The surface of Cu_2+1_O-coated Si electrode is smooth and integrated with only some unconspicuous cracks after 100 cycles (Fig. 7a). And the cross section of Cu_2+1_O-coated Si electrode is keeping entirely integrated (Fig. 7c). For comparison, pristine Si electrode becomes extremely rough and has obvious cracks both in the surface (Fig. 7b) and the cross section images (Fig. 7d). We believe that the Cu_2+1_O coating layer suppress the volume changes of Si during insertion/extraction processes of lithium ions. It is helpful for the electrode keeping integrated and electrical contact during long cycles [24, 25].

## Conclusions

The coating of Cu_2+1_O on Si particles prepared by simple hydrolysis reaction remarkably improved the cycle performance of the battery with an initial reversible capacity of 3063 mAh/g and a CE of 82.9 %. The reversible capacity of the composite remained as 1923.5 mAh/g, 62.8 % of its initial capacity after 100 cycles and 1060 mAh/g after 350 cycles, corresponding to a CE of ≥99.8 % with a current density of 300 mA/g. The composite is easy for mass production. The corresponding electrode maintained extremely good structure and had excellent electrochemical performance during cycling. This is attributed to the enhanced conductivity and elasticity benefited from the Cu_2+1_O coating layer.

## Abbreviations

CE: coulombic efficiency
CV: cyclic voltammetry
EIS: electrochemical impedance spectroscopy
HF: hydrofluoric acid
SEI: solid electrolyte interface
TEM: transmission electron microscope
XRD: X-ray diffraction
